# Genome Sequence of a SARS-CoV-2 VUI 202012/01 Strain Identified from a Patient Returning from London, England, to the Apulia Region of Italy

**DOI:** 10.1128/MRA.01487-20

**Published:** 2021-01-28

**Authors:** Daniela Loconsole, Anna Sallustio, Marisa Accogli, Francesca Centrone, Loredana Capozzi, Laura Del Sambro, Antonio Parisi, Maria Chironna

**Affiliations:** a Department of Biomedical Sciences and Human Oncology-Hygiene Section, University of Bari, Bari, Italy; b Azienda Ospedaliero Universitaria Consorziale Policlinico, Bari, Italy; c Istituto Zooprofilattico Sperimentale della Puglia e della Basilicata, Foggia, Italy; DOE Joint Genome Institute

## Abstract

The coding-complete sequence of severe acute respiratory syndrome coronavirus 2 (SARS-CoV-2) was obtained from a sample from a 25-year-old female returning to the Apulia region of Italy from England. The characterized strain showed all of the spike protein mutations defining SARS-CoV-2 VUI 202012/01, as well as other mutations in the spike protein and in other genomic regions.

## ANNOUNCEMENT

The ongoing pandemic of coronavirus disease 2019 (COVID-19) is caused by severe acute respiratory syndrome coronavirus 2 (SARS-CoV-2), an RNA virus that belongs to the genus *Betacoronavirus* in the family *Coronaviridae*. On 20 December 2020, the European Centre for Disease Prevention and Control (ECDC) signaled a rapid increase in the spread of a SARS-CoV-2 variant in the United Kingdom (1). This variant, called SARS-CoV-2 VUI 202012/01, is characterized by multiple spike protein mutations resulting in a significant increase in transmissibility compared to previously circulating variants (1). Poor clinical outcomes and higher mortality rates have not been reported for people infected with SARS-CoV-2 VUI 202012/01 (1, 2). However, due to the higher reproduction number, countries are requested to signal the circulation of the variant. Timely reporting is crucial to stress the importance of avoiding nonessential travel and social activities, particularly in a period of the year characterized by family and social mixing, since the possible spreading of SARS-CoV-2 VUI 202012/01 represents a major concern.

Here, we announce the draft genome sequence of a strain of SARS-CoV-2 VUI 202012/01 that was identified from a nasopharyngeal swab specimen collected on 19 December 2020 from a 25-year-old woman returning to the Apulia region of Italy from London, England. The approval by a research ethics committee for sequencing of the strain was not required because the activities described were conducted as part of the legislated mandate of the Health Promotion and Public Health Department of Apulia. All procedures were carried out in accordance with the Declaration of Helsinki, as revised in 2013, for human subjects. The RNA was extracted using the MagMAX viral/pathogen nucleic acid isolation kit (Thermo Fisher Scientific). The molecular test was performed using a three-target (N, ORF1ab, and S) assay from Thermo Fisher Scientific (TaqPath reverse transcription real-time PCR COVID-19 assay), and the sample showed the spike gene target failure (SGTF) but was positive for the other two targets. The sample also was positive with the SARS-CoV-2 antigenic test (Lumipulse SARS-CoV-2 Ag kit; Fujirebio).

Whole-genome sequencing was performed with the nasopharyngeal swab sample using the Ion Torrent platform (Thermo Fisher Scientific). The library was prepared with the Ion AmpliSeq Plus library kit according to the manufacturer’s instructions, using Ion AmpliSeq SARS-CoV-2 RNA custom primers (Thermo Fisher Scientific).

Quality control of AmpliSeq reads, as well as alignment of the reads to the SARS-CoV-2 isolate Wuhan-Hu-1 complete genome, was performed within the Torrent Server of the Ion Torrent S5 sequencer using default settings. The aligned reads were utilized for both reference-guided assembly and variant calling. The quality metrics for the reference-based assemblies are as follows: 784,477 sequence reads (average length, 149 bp), 674,160 mapped reads, GC content of 40%, and average base coverage depth of 3,222×. The total size of the genome was 29,780 bp. Assembly was performed with the Iterative Refinement Meta-Assembler (IRMA) v.1.3.0.2 (3), which produced a consensus sequence for this sample using a >50% cutoff value for calling single-nucleotide polymorphisms.

**FIG 1:**
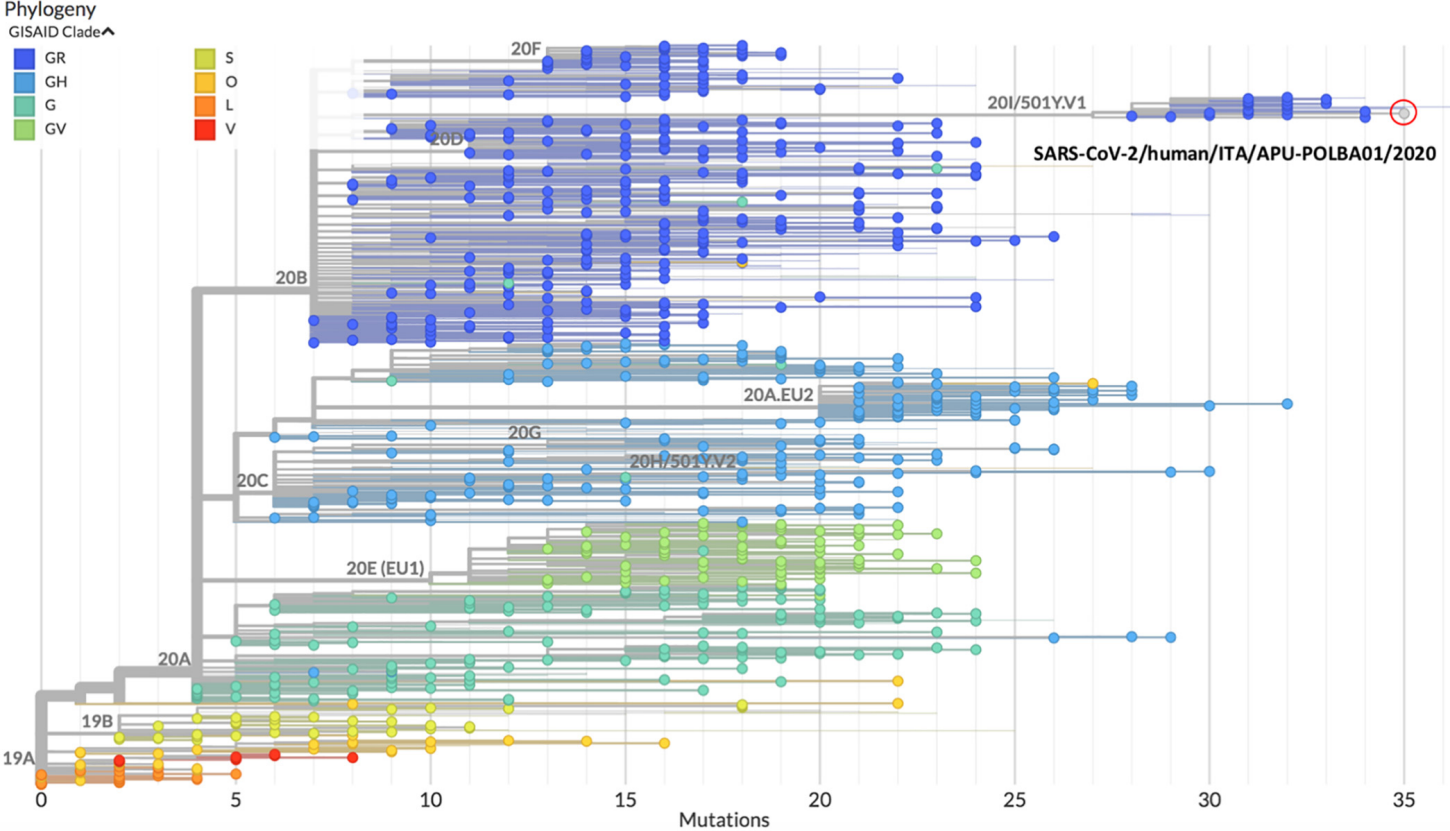
Phylogenetic analysis of the SARS-CoV-2/human/ITA/APU-POLBA01/2020 genome sequence. Other genomes were retrieved from GISAID (https://www.gisaid.org) on 8 January 2021. The colors of the dots are classified according to the GISAID clades. Nextstrain clades are indicated at the branches.

The phylogenetic analysis was performed by using the Nextclade sequence analysis Web app (https://clades.nextstrain.org) (4). The mutations in the strain are listed in Table 1. Figure 1 shows the genetic relationship between SARS-CoV-2/human/ITA/APU-POLBA01/2020 and other strains in the GISAID database. The strain was grouped in clade GR and showed all of the spike protein mutations defining SARS-CoV-2 VUI 202012/01 (1, 2), as well as other mutations in other genomic regions.

**TABLE 1:** List of mutations of the strain SARS-CoV-2/human/ITA/APU-POLBA01/2020

Region	Mutation(s)
Spike	A570D, D614G, D1118H, H69del, N501Y, P681H, S982A, T716I, V70del, Y145del
N	D3L, G204R, R203K, S235F
NS8	Q27stop, R52I, Y73C
NSP2	G465S
NSP3	A890D, I1412T, T183I
NSP6	S106del, G107del, F108del
NSP12	P323L

This genome indicates that the “UK variant” SARS-CoV-2 VUI 202012/01 is present in the Apulia region. The SGTF could be considered a proxy for the carriage of SARS-CoV-2 VUI 202012/01 (2). This is an important point, since it could work as a screening measure for the timely identification of potential carriers of the variant in order to perform rapid genetic characterization of the strains. The possible finding of the local spread of such a variant is worrisome. Strict molecular surveillance to identify this variant in sporadic cases or clusters is thus urgently needed.

### Data availability.

This whole-genome sequence has been deposited in GenBank as SARS-CoV-2/human/ITA/APU-POLBA01/2020 under the accession number MW450666; the SRA accession number is SRR13416427. The GISAID accession number for the sequence is EPI_ISL_735504 (www.gisaid.org).
